# Case Report: The deceptive vein — when benign-appearing laryngeal lesions mask primary laryngeal classic Kaposi sarcoma

**DOI:** 10.3389/fonc.2026.1863012

**Published:** 2026-08-28

**Authors:** Feras Eleyan, Nasser Abualia, Rama Shraim, Mohamad Shraim, Akram Karama, Abdallah Altell, Mohand Abulihya

**Affiliations:** 1 Department of Oncology, Hadassah Medical Center, Jerusalem, Israel; 2 Department of Oncology, Istishari Arab Hospital, Ramallah, Palestine; 3 Department of Pathology, Beit Jala Governmental Hospital, Bethlehem, Palestine; 4 Faculty of Medicine, Al-Quds University, Abu Dis, Palestine; 5 Faculty of Applied Science, Palestine Ahliya University, Bethlehem, Palestine; 6 Department of Hematology, Beit Jala Governmental Hospital, Bethlehem, Palestine; 7 Department of Pathology, Istishari Arab Hospital, Ramallah, Palestine

**Keywords:** angiosarcoma, case report, HIV-negative, Kaposi sarcoma, laryngeal mass, vascular spindle cell lesion

## Abstract

**Introduction:**

Primary laryngeal Kaposi sarcoma (KS) is exceptionally rare in HIV-negative, with only 20 reported cases. The diagnosis is challenging as it may histologically mimic benign vascular lesions in early-stage KS, which leads to delayed diagnosis. We present a case of a classic KS with primary laryngeal involvement in an HIV-negative male. highlighting the diagnostic pitfalls and the critical role of HHV-8 immunohistochemistry.

**Case presentation:**

A male patient in his sixties with a 40-pack-year smoking history presented with progressive hoarseness and nasal obstruction. Six years earlier, he had been diagnosed with a laryngeal lymphangioma, and a subsequent supraglottic lesion six years later was diagnosed as hemangioma, both with excisional biopsy. Several months later he developed dysphagia and diffuse cervical, axillary, and mediastinal lymphadenopathy. MRI revealed a 5.7 cm ill-defined left supraglottic mass with heterogeneous enhancement and necrosis. Biopsy of the laryngeal lesion and a cervical lymph node demonstrated spindle cell proliferation with positive staining for CD31, CD34, D2-40, and HHV-8, confirming the diagnosis of Kaposi sarcoma. HIV testing was negative. Subsequent examination revealed new violaceous nodular lesions on both lower extremities. He received local radiotherapy and systemic liposomal doxorubicin. Six months after treatment, he developed progressive dysphagia and hoarseness due to radiation-induced fibrosis, not recurrence. His course was complicated by recurrent aspiration pneumonia, leading to septic shock and death.

**Conclusion:**

Primary laryngeal Kaposi sarcoma can occur in HIV-negative patients and may be misdiagnosed as benign vascular lesions such as lymphangioma or hemangioma. A high index of suspicion is warranted for any vascular spindle cell lesion of the larynx, regardless of HIV status. HHV-8 immunohistochemistry is an essential confirmatory tool that should be performed early, even when initial biopsies appear benign.

## 1 Introduction

Kaposi Sarcoma (KS) was initially described by Dr. Moritz Kaposi in 1872 as a rare, idiopathic brownish-red to bluish-red cutaneous nodules. KS presents mostly as a multifocal low-grade vascular neoplasm affecting the skin of the lower extremities (1). The global incidence of KS in 2020 was approximately 0.39 cases per 100,000 people (2). KS has a higher incidence among patients infected with HIV (3).

KS may affect any organ in the human body, including the skin, lymph nodes, mucous membranes, and internal organs such as the lungs and gastrointestinal tract. Its presence in the head and neck is not uncommon. However, laryngeal involvement is relatively rare, particularly among immunocompetent individuals (4). This report describes a case of KS with laryngeal involvement as the initial presentation in an HIV-negative patient. The objective is to increase awareness of this rare manifestation and emphasize the importance of considering it in the differential diagnosis of laryngeal vascular lesions, even among immunocompetent patients.

## 2 Case presentation

A male patient in his sixties presented with progressive hoarseness of six months’ duration accompanied by symptoms of nasal obstruction. He reported a 40-pack-year smoking history and a past medical history significant for a laryngeal mass diagnosed histologically as lymphangioma six years earlier. He had no history of corticosteroid use, immunosuppression, recurrent infections, or diabetes mellitus. Examination revealed dry nasal mucosa, left-sided secretory otitis media, with no cervical lymphadenopathy. Nasopharyngoscopy revealed a round nasopharyngeal mass, which was confirmed on CT as a soft-tissue lesion extending into the sphenoid and left maxillary sinuses with an associated air–fluid level. Flexible laryngoscopy demonstrated a supraglottic cystic lesion limiting vocal cord mobility for which an excisional biopsy revealed a hemangioma without evidence of malignancy, while transnasal nasopharyngeal biopsies revealed reactive lymphoepithelial tissue. The patient subsequently underwent adenoidectomy.

Over the next 6 months, the patient experienced a recurrence of hoarseness accompanied by new-onset dysphagia. During that period, he had recurrent upper respiratory tract infections treated with antibiotics and analgesics, with minimal sustained response. At this stage, chest CT revealed bilateral cervical and axillary lymphadenopathy, the largest right axillary node measuring 1.4 × 1.3 cm, in addition to mediastinal lymphadenopathy, with the largest mediastinal node measuring 2.1 × 1.1 cm.

**Figure 1:**
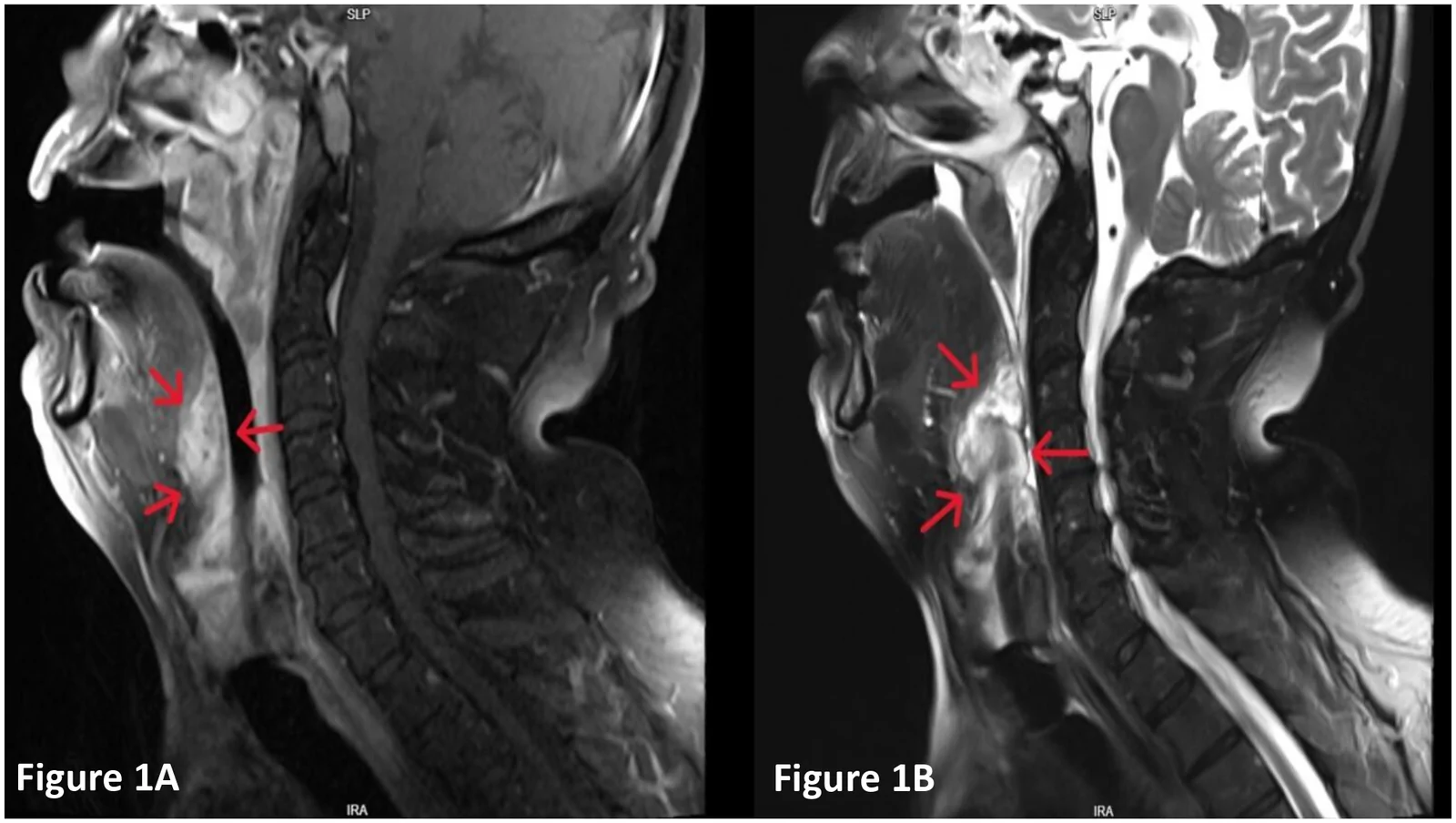
Contrast-enhanced neck MRI. **(A)** T1-weighted image showing hypointensity with diffusion restriction, heterogeneous enhancement, and focal areas of necrosis. **(B)** T2-weighted image showing a left supraglottic mass with heterogeneous hyperintensity.

Given the patient’s prior medical history and recent dysphagia with lymphadenopathy, contrast-enhanced neck MRI was performed, revealing an ill-defined laryngeal mass in the left supraglottic space, extending from the level of the false vocal cord through the aryepiglottic folds, with associated thickening of the aryepiglottis. The lesion measured approximately 5.7 × 2.7 × 5.0 cm, showed heterogeneous signal intensity with diffusion restriction, T1 hypointensity and T2 hyperintensity (**Figures 1A, B**), and demonstrated heterogeneous enhancement with focal areas of necrosis.

Subsequently, flexible bronchoscopy was performed, revealing extensive edema and hyperemia of the supraglottic region with near-complete obstruction. The arytenoids and vocal cords showed mucosal irregularities and nodularity, while the trachea and bronchi appeared normal. Multiple biopsies were obtained from the supraglottic area and vocal cords, complicated by bleeding, which was controlled. A tracheostomy was performed at the end of the procedure to secure the airway in view of edema and the highly vascular nature of the lesion. During the same procedure, an excisional biopsy of the right lower cervical lymph node was performed.

**Figure 2:**
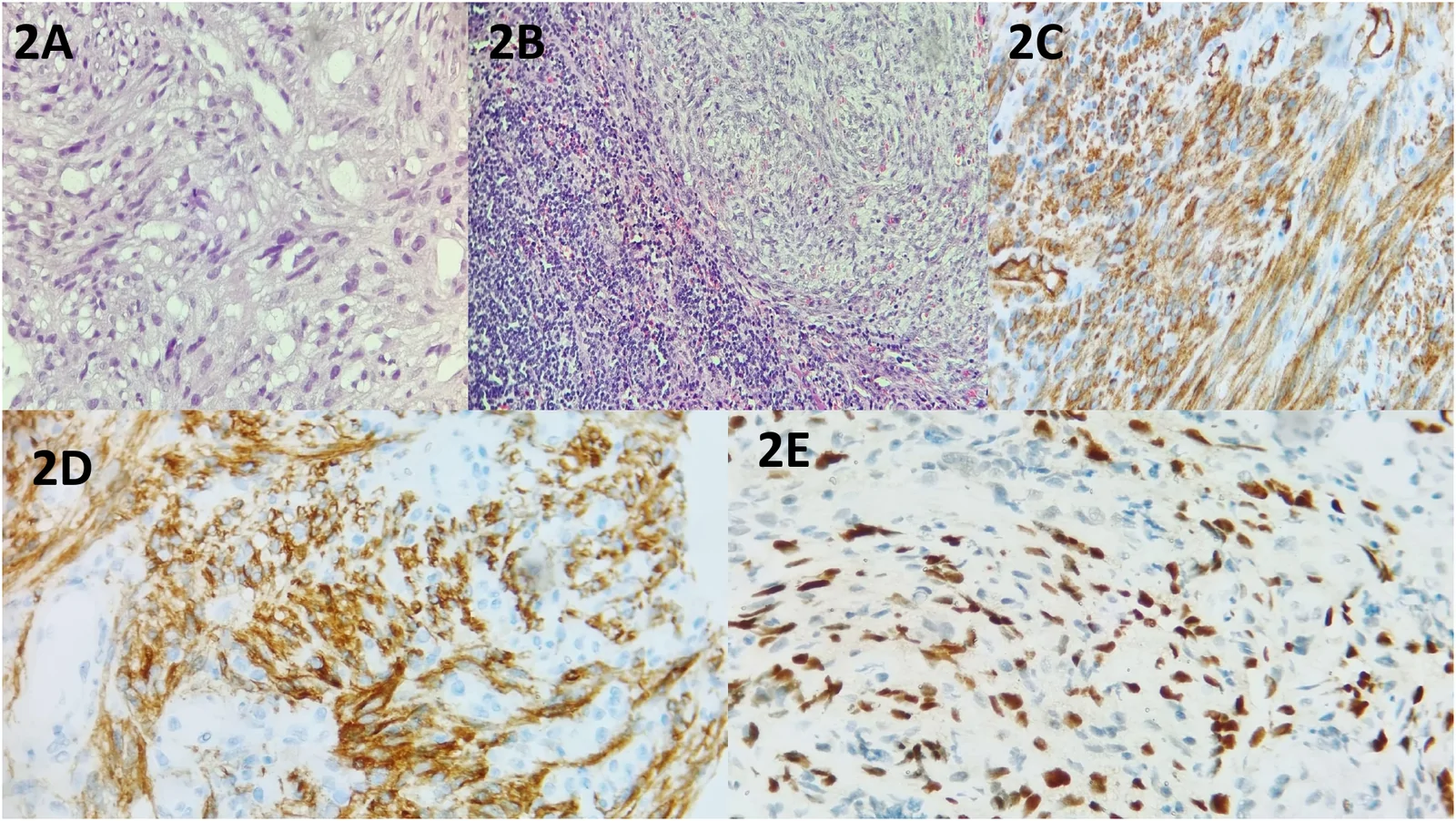
Histopathological and immunohistochemical examination of the laryngeal mass and cervical lymph node biopsy. **(A)** Hematoxylin and eosin (H&E) stain of the laryngeal mass showing spindle cell proliferation. **(B)** H&E stain of a cervical lymph node showing involvement by the same spindle cell proliferation. **(C)** Positive CD 31 immunohistochemical staining. **(D)** Positive D2–40 immunohistochemical staining. **(E)** Positive HHV-8 immunohistochemical staining.

Pathological evaluation of the laryngeal lesion demonstrated spindle cell proliferation (**Figure 2A** and was similarly noted in a biopsy of an enlarged right lower cervical lymph node (**Figure 2B**).

Immunohistochemistry revealed tumor cell positivity for CD31, D2-40, and HHV-8 (**Figures 2C–E**), with negative staining for Pan-Cytokeratin (AE1/AE3), EMA, Desmin, S100, SOX10, SMA, CK5/6, P63, DOG1, CD99 and STAT6, confirming the diagnosis of multicentric Kaposi sarcoma. Subsequent HIV testing was negative.

Following the diagnosis, extensive clinical examination revealed new-onset multiple violaceous nodular lesions on both lower extremities that were not apparent on previous examination. Given the disseminated nature of the disease, the patient was treated with local laryngeal radiotherapy and systemic chemotherapy with liposomal doxorubicin for 6 cycles. He showed significant regression of his laryngeal and cutaneous lesions, which is evident on PET scan after the third cycle.

Six months after completing treatment, the patient developed progressive hoarseness and dysphagia; PET scan showed no evidence of tumor recurrence, and his symptoms were attributed to late complications of local radiotherapy, involving laryngeal and pharyngeal fibrosis and edema. A PEG tube was placed for nutritional support, and a tracheostomy was reinserted due to airway compromise.

His course was complicated by recurrent aspiration pneumonia, and despite treatment with broad-spectrum antibiotics and intensive supportive care, he progressed to septic shock with hypotension and multi-organ failure. Despite maximal resuscitation efforts, he ultimately passed away.

## 3 Discussion

Kaposi Sarcoma (KS) is a cutaneous vascular neoplasm that presents in four epidemiologic forms: Classic, African endemic, epidemic AIDS-related, and iatrogenic KS (5). These forms vary in clinical severity, ranging from slowly growing lesions of the classic type to the rapidly progressive epidemic type (6).

It is established that KS is caused by an infection with Human herpesvirus-8 (HHV-8), also called Kaposi sarcoma herpesvirus (KSHV) (5). HHV8 is a DNA virus that was first described in 1994 by Chang et al., who identified it in tissues of patients suffering from AIDS/KS. Other factors have been implicated in KS pathogenesis; the most evident of them is immunosuppression. Host genetic factors are presumed to play a role also; for example, classic KS has been correlated to specific gene polymorphisms, including diplotypes of interleukin-8 receptor-β, IL-13, and particular human leukocyte antigen (HLA) haplotypes (3).

Primary Kaposi sarcoma of the larynx is rare (7). While approximately 80 total cases of laryngeal KS have been reported (8, 9), only 20 of these cases occurred in HIV-negative patients (10, 11), making our case exceptionally rare, demonstrating classic KS presenting primarily in the larynx of an HIV-negative patient. Classic KS predominantly affects elderly males and follows an indolent course (12). Symptoms may include hoarseness, dysphagia, dysphonia, hemoptysis, and airway obstruction (7, 13, 14). Our patient similarly had an indolent course of recurrent laryngeal lesions and presented with hoarseness and dysphonia.

Cross-sectional imaging, including magnetic resonance imaging (MRI), is typically performed initially to evaluate and localize laryngeal lesions. However, the definitive diagnosis of laryngeal KS requires biopsy despite the high risk of bleeding due to the tumor’s vascular nature (15). Endoscopy with Narrow Band Imaging (NBI) is essential for guiding the biopsy and minimizing this risk.

NBI helps assess a lesion’s vascularity and can guide the endoscopist towards a total excision rather than an incisional biopsy (9). Many authors recommend a prophylactic tracheostomy due to the high risk of acute upper airway obstruction from edema or bleeding (16).

In our case, NBI was unavailable at our facility. In addition, the lesion manifested as mucosal nodules without a discrete mass, making total excision unfeasible. The procedure was complicated by bleeding, which necessitated tracheostomy.

Histopathology examination of KS reveals uniform spindle cells in a background of slit-like vascular spaces and lymphocytic infiltrates. However, the disease encompasses a spectrum of histological types ranging from well differentiated to poorly differentiated. The diagnostic challenge lies in the early, well-differentiated KS, where biopsy specimens often lack the distinctive feature of KS and may instead resemble other vascular tumors such as lymphangioma or hemangioma (6, 17).

Despite their morphological differences, early KS can resemble lymphangioma and potentially delay the diagnosis. This is demonstrated by reports of lymphangioma-like KS, where up to half of the lesional tissue is composed of anastomosing, ectatic lymphatic vessels surrounded by spindle cells of KS. This diagnostic challenge may arise due to incomplete tissue sampling (18).

Similarly, early KS may also be mistaken for hemangioma. Ectatic vessel pattern of KS is composed of large blood-filled channels lined by an endothelial layer resembling hemangioma (19).

In order to overcome this challenge, adequate sampling of such lesions and careful histological assessment are essential, especially in the context of the patient’s immune status and demographics, in order to detect KS-specific features: spindle cell proliferation, red blood cell extravasation, and hemosiderin pigment deposition (17).

Our patient initially had a laryngeal lesion diagnosed as lymphangioma at another center. Followed by a recurrence after several years, and was diagnosed as hemangioma before the final, definitive diagnosis of KS was made. Given the several recurrences despite treatment, it is possible that the lesion was KS all along. However, confirming this hypothesis requires clonality testing to be proven.

Immunohistochemistry helps distinguish KS in such cases, as the tumor cells express CD34, CD31, and D2-40, while markers for Cytokeratins, EMA, Desmin, S100, and SMA are typically negative (6). However, this immunoprofile can also overlap with other vascular spindle cell lesions, most notably low-grade angiosarcoma, which is a critical differential diagnosis distinguished by its greater degree of cellular atypia. Moreover, both KS and angiosarcoma can involve lymph nodes. Angiosarcoma metastasizes to regional nodes in about 20% of cases (20), whereas KS often presents with multifocal systemic lymphadenopathy reflecting its disseminated nature (21). In this case, both KS and angiosarcoma were considered in the differential diagnosis, as both can involve lymph nodes and share a similar immunohistochemical profile.

HHV-8 immunohistochemistry provides the solution, as it has been proven to be the most useful confirmatory tool for KS and for ruling out angiosarcoma and other vascular mimickers (22, 23). Accordingly, HHV-8 staining was effectively utilized in our case, demonstrating strong positivity in both the laryngeal and cervical lymph node lesions and thereby securing the diagnosis.

Treatment of laryngeal KS depends on many factors, including the epidemiologic type of KS, particularly whether it is HIV-related or not, and the extent of disease. Classic KS is a highly radiosensitive tumor, and low-dose local radiotherapy has been demonstrated to achieve complete remission (24), and it is recommended after excision of the laryngeal lesions (14). However, laryngeal radiation has been associated with early and late complications, some of which may be life-threatening. Radiotherapy causes local hypoxia, stimulates tissue fibrosis, endarteritis, and tissue edema and mucositis. These consequences may ultimately lead to chondroradionecrosis (25). These potential complications should be carefully weighed against the favorable response of KS to low-dose radiation, ensuring the treatment strategy balances disease control with functional preservation. Many other modalities have been used, for example, intralesional chemotherapy and laser ablation (26).

For multicentric disease, it is recommended to use systemic chemotherapy; Vincristine, Vinblastine, Bleomycin, and Etoposide have shown activity against KS (14, 26). Currently, the mainstay of systemic agents for KS treatment is liposomal anthracyclines (pegylated liposomal doxorubicin and liposomal daunorubicin) and taxanes (paclitaxel) (27). These modalities have shown effectiveness in controlling such cases.

Our patient was not a candidate for tumor resection due to the absence of an appreciable mass. He received local low-dose radiotherapy, and in the context of his systemic illness, he received liposomal doxorubicin. He showed significant regression of his laryngeal and cutaneous lesions.

## 4 Conclusion

Primary laryngeal Kaposi sarcoma in HIV-negative patients is rare and easily mistaken for benign vascular lesions. This case highlights the diagnostic value of HHV-8 immunohistochemistry in any vascular spindle cell lesion of the larynx, even when initial biopsies suggest benign pathology such as lymphangioma or hemangioma. A high index of suspicion and repeat biopsy with HHV-8 staining are essential for timely diagnosis and appropriate management.

### 4.1 Take-home messages

• Vascular spindle cell lesions can share similar histomorphology and sometimes similar immunohistochemistry profile; however, they differ substantially in management. Accurate differentiation is therefore crucial.
• Kaposi sarcoma should be considered in the differential diagnosis of vascular spindle cell lesions, especially in immunocompromised patients, those of Mediterranean descent, or in cases with concerning features such as recurrence despite treatment. HHV-8 immunohistochemistry is essential for definitive diagnosis.
• Benign-appearing lesions such as lymphangioma or hemangioma may represent early well-differentiated Kaposi sarcoma; repeat biopsy with HHV-8 staining is warranted when the clinical course is atypical.
• Narrow Band Imaging (NBI) is recommended for laryngeal lesions to guide the biopsy approach and reduce the risk of bleeding.

## Data Availability

The datasets presented in this article are not readily available because no separate datasets were generated beyond the clinical, histopathological, immunohistochemical, and imaging data already fully presented within the manuscript text and figures. Therefore, no restrictions apply as there are no additional raw datasets to share. Requests to access the datasets should be directed to Requests for additional data can be directed to the corresponding author: Akram Karama at Akkrama@yahoo.com. However, no separate raw datasets exist beyond what is presented in the manuscript.
